# Intratumoral heterogeneity characterized by pretreatment PET in non-small cell lung cancer patients predicts progression-free survival on EGFR tyrosine kinase inhibitor

**DOI:** 10.1371/journal.pone.0189766

**Published:** 2018-01-31

**Authors:** Sehhoon Park, Seunggyun Ha, Se-Hoon Lee, Jin Chul Paeng, Bhumsuk Keam, Tae Min Kim, Dong-Wan Kim, Dae Seog Heo

**Affiliations:** 1 Department of Internal Medicine, Seoul National University Hospital, Seoul, Republic of Korea; 2 Department of Nuclear Medicine, Seoul National University Hospital, Seoul National University Hospital, Seoul, Republic of Korea; 3 Division of Hematology/Oncology, Department of Medicine, Samsung Medical Center, Sungkyunkwan University School of Medicine, Seoul, Republic of Korea; Wayne State University, UNITED STATES

## Abstract

Intratumoral heterogeneity has been suggested to be an important resistance mechanism leading to treatment failure. We hypothesized that radiologic images could be an alternative method for identification of tumor heterogeneity. We tested heterogeneity textural parameters on pretreatment FDG-PET/CT in order to assess the predictive value of target therapy. Recurred or metastatic non-small cell lung cancer (NSCLC) subjects with an activating *EGFR* mutation treated with either gefitinib or erlotinib were reviewed. An exploratory data set (n = 161) and a validation data set (n = 21) were evaluated, and eight parameters were selected for survival analysis. The optimal cutoff value was determined by the recursive partitioning method, and the predictive value was calculated using Harrell’s C-index. Univariate analysis revealed that all eight parameters showed an increased hazard ratio (HR) for progression-free survival (PFS). The highest HR was 6.41 (*P*<0.01) with co-occurrence (Co) entropy. Increased risk remained present after adjusting for initial stage, performance status (PS), and metabolic volume (MV) (aHR: 4.86, *P*<0.01). Textural parameters were found to have an incremental predictive value of early EGFR tyrosine kinase inhibitor (TKI) failure compared to that of the base model of the stage and PS (C-index 0.596 vs. 0.662, *P* = 0.02, by Co entropy). Heterogeneity textural parameters acquired from pretreatment FDG-PET/CT are highly predictive factors for PFS of EGFR TKI in EGFR-mutated NSCLC patients. These parameters are easily applicable to the identification of a subpopulation at increased risk of early EGFR TKI failure. Correlation to genomic alteration should be determined in future studies.

## Introduction

Although non-small cell lung cancer (NSCLC) is a leading cause of cancer-related death and comprises 23% of total cancer deaths[1], a subpopulation with activating epidermal growth factor receptor (EGFR) mutations have demonstrated prolonged progression-free survival (PFS) with the development of EGFR tyrosine kinase inhibitors (TKI)[2–4]. However, target therapies which focus on a critical survival pathway do not benefit all patients. This phenomenon may be partially explained by intratumoral heterogeneity, which refers to the existence of subpopulations of distinct cancer cells within a tumor[5]. For this reason, it has been a research focus[6] in this current era of target therapy[7–10]. Moreover, a small population of sub-clone with genetic heterogeneity remains challenging to identify.

Due to the disadvantages of executing multiple biopsies and the high cost of genomic evaluation, alternative approaches to detect intratumoral heterogeneity through non-invasive imaging have been investigated[11], and attempts to determine genomic variation by interpreting large amounts medical imaging data have been conducted[12]. Conventional positron emission tomography/computed tomography (PET/CT) indices, such as average standardized uptake value (SUV_average_) and maximum standardized uptake value (SUV_max_), are also used as parameters of inter-tumor heterogeneity[13, 14]. By extension, metabolic heterogeneity characterized by local and regional textural parameters by 2[18F] fluoro-2-deoxy-_D_-glucose (FDG) uptake in pretreatment FDG-PET/CT allows the prediction of chemotherapeutic response[15, 16], disease progression after concurrent chemoradiotherapy[17], and overall survival[18–20]. Moreover, these parameters have demonstrated significant predictive value in NSCLC patients who have undergone curative resection[21].

To date, despite the clinical importance of identifying intratumoral heterogeneity, limited adjuvant methods have been investigated to predict the response to target therapy. In this study, we assessed the clinical value of local and regional textural parameters from a pretreatment FDG-PET/CT scan of NSCLC patients with activating EGFR mutations undergoing EGFR TKI treatment.

## Patients and methods

### Study population

NSCLC patients (n = 2012) who were treated with either gefitinib or erlotinib from July 2002 to September 2014 in Seoul National University Hospital (SNUH) were reviewed. Subjects who had not been tested for EGFR genotype prior to treatment (n = 1047) and subjects who had been tested but lacked an EGFR mutation (n = 274) were excluded. Inclusion criteria were as follows: (i) subjects with exon 19 deletion or exon 21 point mutation [L858R or L861Q] confirmed either by peptide nucleic acid clamping or by DNA sequencing; and (ii) subjects with pre-EGFR TKI treatment FDG-PET/CT scan available.

**Fig 1:**
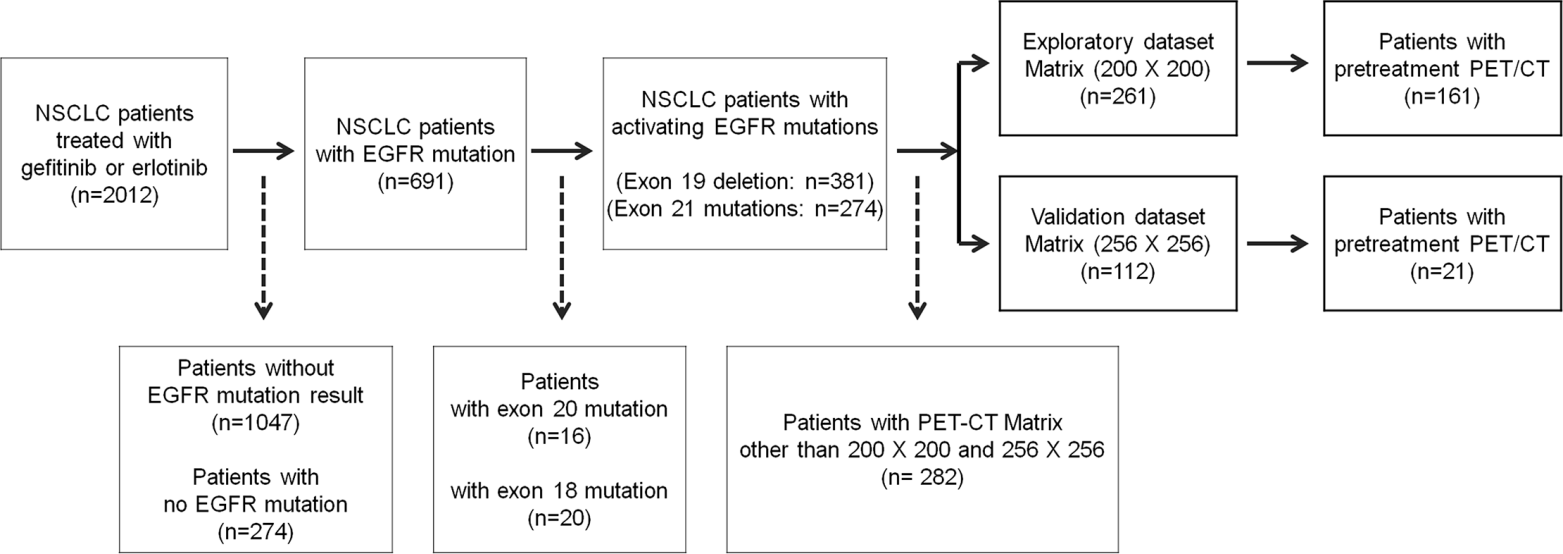
Flow chart of patient selection. Abbreviations: NSCLC = non-small cell lung cancer, EGFR = epidermal growth factor receptor, PET = positron emission tomography, CT = computed tomography.

To avoid any potential bias due to different PET-CT matrix size, we selected 200 × 200 matrix size which contains most number of patients for the exploratory subset (n = 261) and 256 × 256 for the validation subset (n = 112). Subjects with other than above two matrix sizes were excluded from analysis (n = 282). The subjects were further selected based on the availability of pretreatment FDG-PET/CT scan and total of 182 subjects’ data, 200 × 200 matrix size (n = 161) and 256 × 256 matrix size (n = 21), was used for the final analysis. (Fig 1)

### Clinical data collection

Medical history, pathology, and imaging data were reviewed retrospectively. The 7^th^ edition of the American Joint Committee on Cancer Staging manual was used to define initial stage, and treatment response was evaluated by comparing post-treatment CT to pretreatment CT in accordance with the Response Evaluation Criteria in Solid Tumor (RECIST) ver. 1.1[22]. Survival data were collected from the Korean death registry. All data were acquired under the supervision of the Institutional Review Board of SNUH (SNUH IRB No.1411-026-623). This study is classified as a retrospective observational study which IRB waives patient permission for the review of the de-identified medical record.

### FDG-PET/CT imaging protocol

After 6 h of fasting, FDG-PET/CT images were acquired using dedicated FDG-PET/CT scanners (Biograph 40 mCT, Biograph 64 mCT and Biograph TruePoint; Siemens, Erlangen, Germany). One hour prior to scanning, 5.18 MBq/kg of ^18^F-FDG was injected intravenously. The fasting blood glucose level was maintained at ≤7.8 mmol/L. A pre CT scan was obtained for attenuation correction prior to a PET scan. An ordered subset-expectation maximization algorithm was used for reconstruction and detailed settings were: (i) for Biograph 40 (n = 78) and Biograph 64 (n = 83) mCT scanners, 200 × 200 matrix, time-of-flight, 2 iterations and 21 subsets were adapted; (ii) for Biograph TruePoint scanner (n = 21), 256 × 256 matrix, 4 iterations and 8 subsets were adapted.

### FDG-PET/CT image analysis

**Fig 2:**
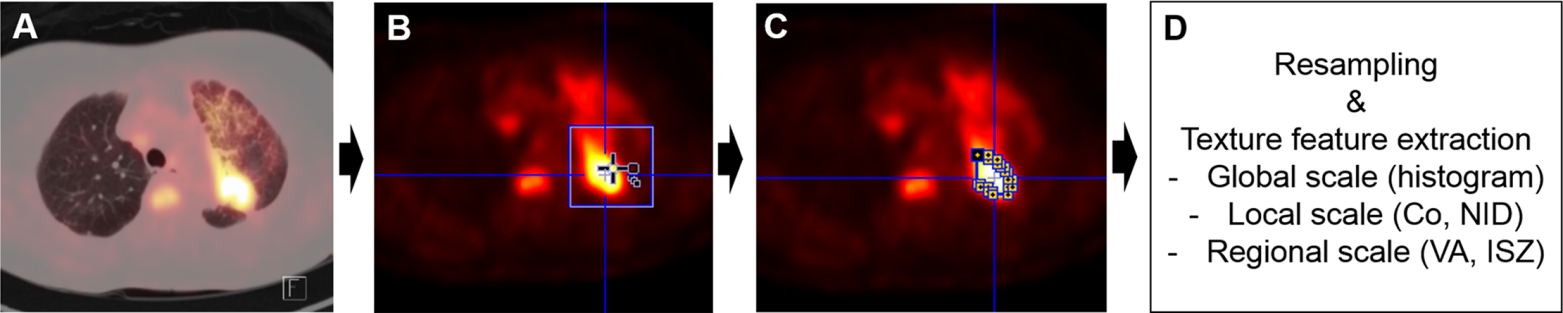
Schematic flow of textural analysis. **(A) FDG-PET/CT scan acquisition. (B) Placement of a volume of interest on the primary tumor. (C) Tumor segmentation by isocontour with SUV of 3.5 (D) Gray scale resampling and texture feature extraction in global, local, and regional scales.** Abbreviations: Co = Co-occurrence, NID = Neighborhood intensity difference, VA = Voxel alignment, ISZ = intensity size zone.

FDG-PET/CT images report were reviewed twice by two different nuclear medicine physicians by one physician validate the other physician’s imaging report. SUV was calculated as the ratio between concentrated radioactivity on the tissue (kBq/mL) and the injected dose per weight (kBq/g). FDG-PET/CT image analysis was conducted by PMOD (PMOD Technologies Ltd., Switzerland) and CGITA v.1.3 software (Chang-Gung Memorial Hospital, Taiwan). The steps for acquisition of heterogeneity textural parameters were: FDG-PET/CT scan acquisition, VOI (volume of interest) placement, tumor segmentation, resampling, and feature extraction by textural analysis (Fig 2). After FDG-PET/CT scan acquisition, a VOI was placed on the primary tumor in most cases. In the eight cases with no available lung mass for analysis, a VOI was placed on metastatic lesions in skeletal regions such as the spine, ribs, and femur. The tumor was segmented with a predetermined cutoff value of SUV 3.5. Subsequently, gray levels of the segmented tumor were resampled to standardize the range of values. This was done to reduce noise in the delineated tumor and to normalize the scales among different cases[15]. Sixty-four gray levels were adapted for an optimal resampling scale in this study. Compared to other sampling scales, this attained higher reproducibility, robustness, and the potential for information complementary to MV[23–25].

### Textural analysis

**Fig 3:**
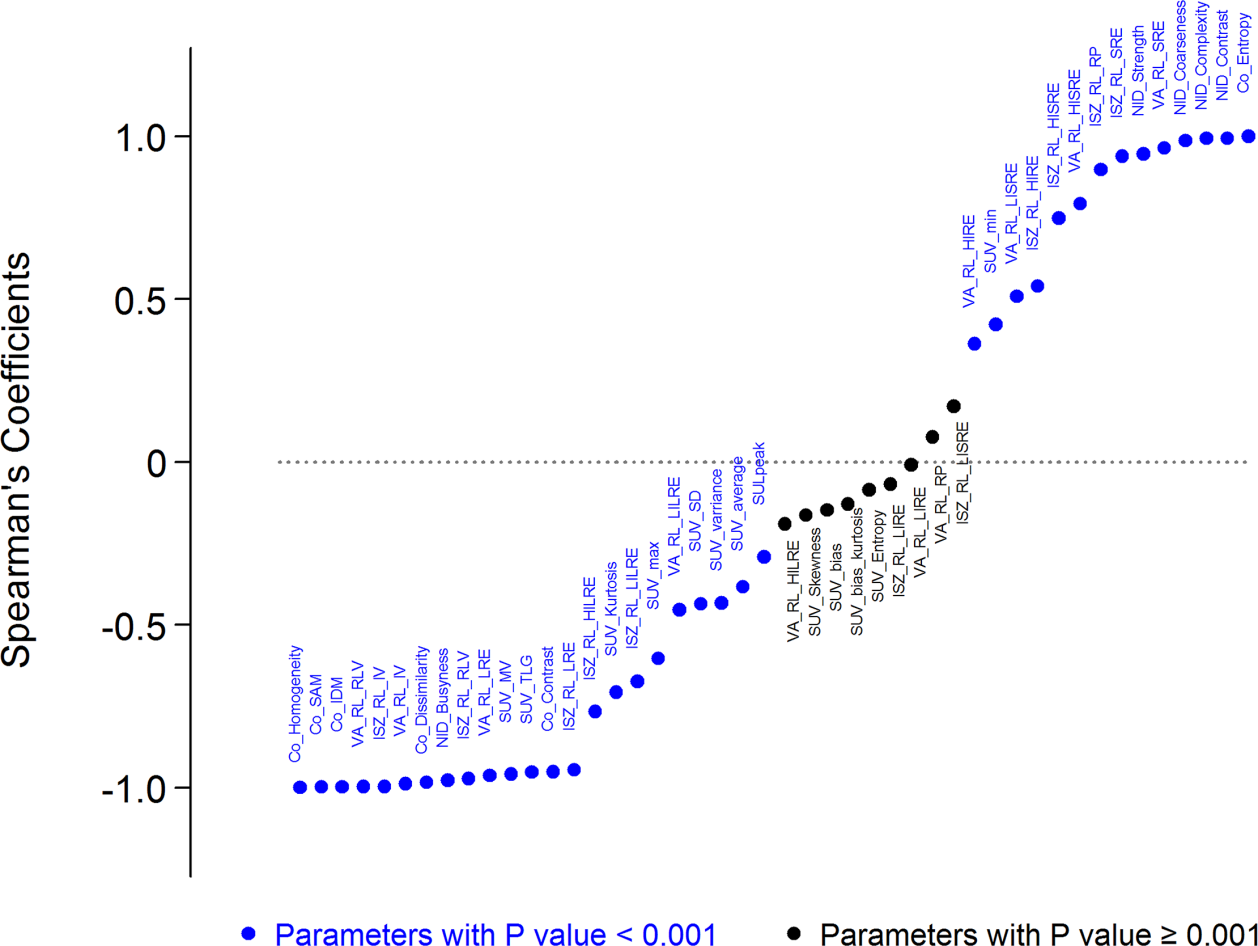
Spearman’s rank correlation coefficients of textural parameters compared to the value of co-occurrence entropy. Abbreviations are listed in S1 Table.

Multiple mathematical models for textural analysis were applied. Based on the scales of analysis, statistics-based texture analyses were composed of global, local, and regional scales[26]. It was unclear which scale was appropriate for representing intratumoral heterogeneity to predict PFS of EGFR TKI in cases of TKI treatment for NSCLC patients. Therefore, we included most of the texture features that had been reported in previous studies to be predictive of treatment response by textural analyses. Histogram-based parameters (global features) and reconstructed matrices, which described the relationship between each of the voxels, were applied to calculate heterogeneity. Co-occurrence (Co) matrix based parameters and Neighborhood intensity difference (NID) matrix based parameters were local scale features used to describe the frequency of certain relationships between two voxels of intensity. Two regional matrices, a voxel alignment (VA) matrix, and an intensity size zone (ISZ) matrix, were used to calculate regional scale parameters in this study. All parameters and their abbreviations are displayed in S1 Table. Detail methods of calculating parameters described in Fig 3, S1 Table and S1 Fig were described in a review article by Cook et al[27].

### Statistical analysis

The baseline demographics of the subjects in the exploratory and validation datasets were analyzed with descriptive statistics. Spearman’s rank correlation coefficients of each textural parameter were calculated by comparing texture parameters to the Co entropy value, which is defined as calculated randomness of voxel intensity and has been proposed as a useful parameter for measurement of intratumoral heterogeneity. A Bonferroni correction was used and parameters with *P* ≤ 0.001 were considered as statistically significant. Four textural parameters from the group with positive correlation to Co entropy and four parameters from the group with negative correlation were selected from different feature parents.

Rather than using defined PFS time point for the evaluation, optimal cutoff values were established by a recursive partitioning method[28], which satisfied the highest hazard ratio with *P* ≤ 0.05 and PFS were calculated for groups below and above cut-off value. Applying optimal cutoff values to the survival analysis, hazard ratios (HR) for PFS were calculated by Cox proportional-hazard regression analyses, and Kaplan-Meier curves were used to portray treatment failure. In this study, we have defined early EGFR TKI failure as the group with shorter PFS using the optimal cutoff values. PFS was calculated from the date of initiation of EGFR TKI treatment to the date of cancer progression or all-cause mortality. Multivariate analysis was performed using parameters satisfying *P* ≤ 0.05 following univariate analysis or parameters considered to be clinically significant. Incremental predictive value of PFS of EGFR TKI failure was determined by comparing Harrell’s C-index to different Cox proportional hazard regression models[29].

Statistical analyses were conducted with STATA version 12.1 software (StataCorp, College station, TX, USA) and R-3.1 for Windows (Ross Ihaka and Robert Gentlemen, University of Auckland, New Zealand). All results with a two-tailed *P* ≤ 0.05 were considered to be significant.

## Results

### Characteristics of the study population

Baseline clinical characteristics of the exploratory and validation datasets are shown in Table 1. In the exploratory dataset, the median age was 66 (range, 36–88), 34.2% were male, and 98.8% were diagnosed with adenocarcinoma. The subjects with initial metastatic disease comprised 85.1% of the exploratory study population and 95.7% of the subjects were treated with gefitinib. A total of 94.4% were treated with EGFR TKI as first-line treatment, and the median time difference pretreatment FDG-PET/CT scan to EGFR TKI treatment was 0.5 (range 0.0–2.7) months in the exploratory dataset and 0.7 (range 0.0–4.0) months in the validation dataset.

**Table 1:** Characteristics of the study population.

		Exploratory dataset	Validation dataset	*P*
	n (%)	n = 161	n = 21	
Age (Years)	median (range)	67 (36–88)	68 (48–82)	0.40
Sex				
	M	55 (34.2)	4 (19.1)	0.22
	F	106 (65.8)	17 (80.9)	
Tumor Cell Type				
	ADC	159 (98.8)	17 (80.9)	<0.01
	Others	2 (1.2)	4 (19.1)	
ECOG PS				
	0 and 1	140 (87.0)	15 (71.4)	0.06
	2, 3 and 4	21 (13.0)	6 (28.6)	
Initial Disease Status				
	Recurred	24 (14.9)	2 (9.6)	0.74
	Metastatic	137 (85.1)	19 (90.4)	
EGFR MT				
	Exon 19	87 (54.0)	18 (85.7)	0.01
	Exon 21	74 (46.0)	3 (14.29)	
EGFR TKI				
	Gefitinib	155 (95.7)	21 (100,0)	1.00
	Erlotinib	7 (4.3)	0 (0.0)	
EGFR TKI treatment				
	1st line	153 (95.0)	18 (85.7)	0.15
	2nd line	8 (5.0)	3 (14.3)	
TKI response				
	CR	3 (1.9)	0 (0.0)	0.28
	PR	126 (78.3)	15 (71.4)	
	SD	24 (14.9)	3 (14.3)	
	PD	7 (4.3)	2 (9.5)	
	N/A	1 (0.6)	1 (4.8)	
Time interval between FDG-PET/CT to treatment (Months)				
	median (range)	0.5 (0.0–2.7)	0.7 (0.0–4.0)	<0.01

Abbreviations: ADC = adenocarcinoma, ECOG PS = Eastern Cooperative Oncology Group Performance Status, EGFR = epidermal growth factor receptor, TKI = tyrosine kinase inhibitor, CR = complete response, PR = partial response, SD = stable disease, PD = progressive disease

### Textural parameters associated with early EGFR TKI failure

**Fig 4:**
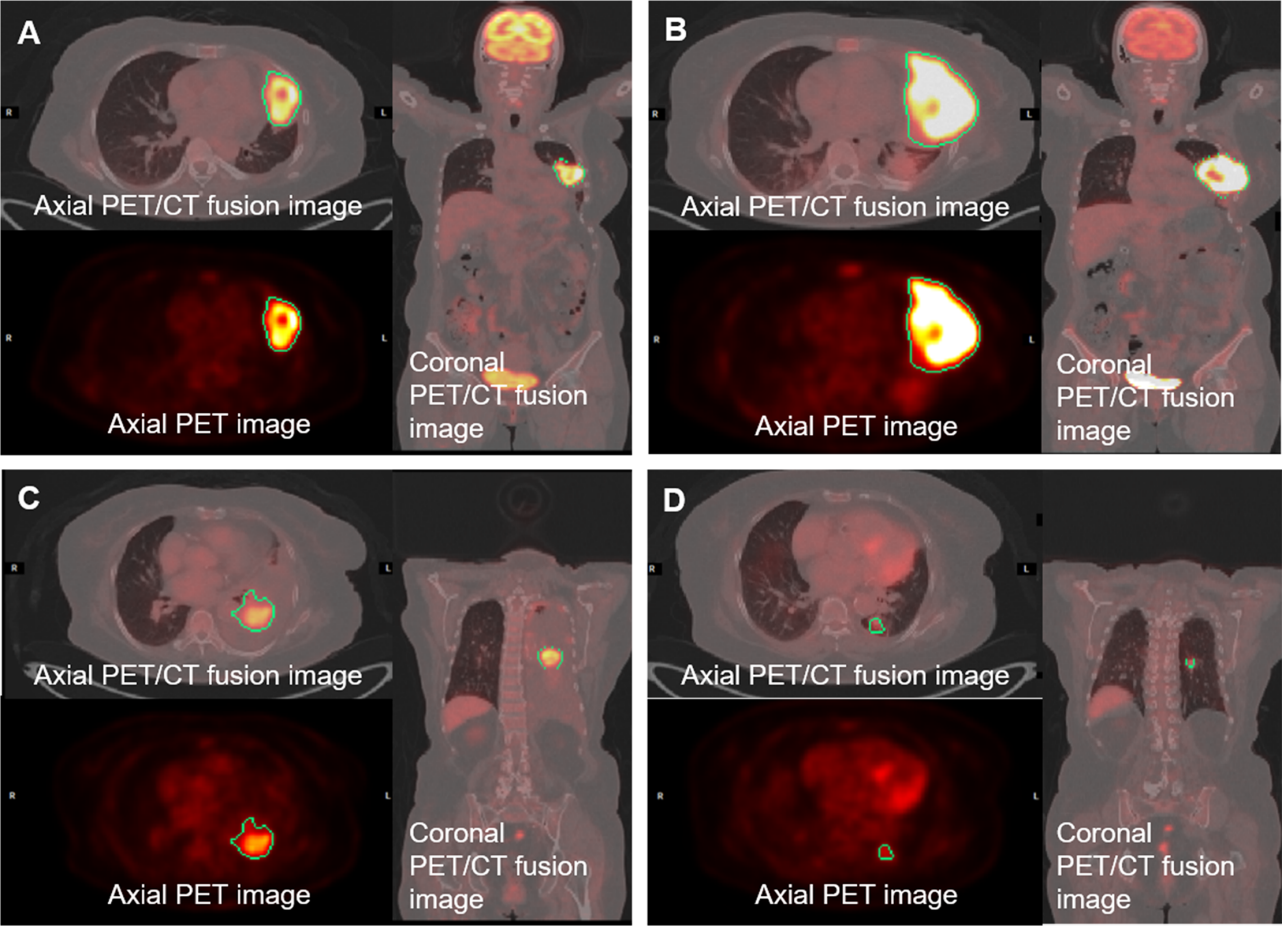
Representative images from the exploratory dataset (200 x 200 matrix). Representative images of two patients who had metabolic volumes with different heterogeneity textural parameters. Patients with increased intratumoral heterogeneity by textural parameters showed poor progression free survival. Panel A & B. A 51-year-old woman with 4.7 months of progression free survival. (A) Pre-treatment FDG-PET/CT images (SUVmax 34.1, metabolic volume 109.3 cm^3^) EGFR exon 19 micro-deletion mutation; Co entropy: -82302; Co homogeneity: 6951; VA intensity variability: 299; VA short run emphasis: 0.458; NID busyness: 1.170; NID contrast: 4.85 x 10–5; ISZ intensity variability: 964.0; and ISZ high intensity zone emphasis: 472.1. (B) Post-treatment FDG-PET/CT images after 4.7 months (SUVmax 36.7, metabolic volume 358.5 cm3) Panel C & D. A 71-year-old woman with 15.4 months of progression free survival. (C) Pre-treatment FDG-PET/CT images (SUVmax 16.0, metabolic volume 95.1 cm^3^) EGFR exon 19 micro-deletion mutation; Co entropy: -53663; Co homogeneity: 5156; VA intensity variability: 680; VA short run emphasis: 0.387; NID busyness: 1.012; NID contrast: 7.98 x 10–5; ISZ intensity variability: 603.8; ISZ high intensity zone emphasis: 530.2. (D) Post-treatment FDG-PET/CT images after 2.8 months (SUVmax: 6.0, metabolic volume: 4.2 cm^3^). Abbreviations: Co = co-occurrence,VA = voxel alignment, NID = neighbor intensity-difference, ISZ = intensity size-zone.

Spearman’s rank correlation coefficients were calculated by comparing textural parameters with Co entropy (Fig 3), and the trend of HR for TKI PFS by binary distribution at the upper and lower 10% and 25% was shown (S1 Fig). Subjects with higher than optimal cutoff values for Co homogeneity, VA intensity variability, NID busyness and ISZ intensity variability, all values whose increase represents increased intratumoral heterogeneity, showed increased HR as PFS of EGFR TKI treatment. Increases in HR were also observed in SUV_max_ (HR: 2.77, 95% confidential interval [CI] 1.58–4.87), SUV_average_ (HR: 1.64, 95% CI 1.01–2.70), and SUV_MV_ (HR: 2.89, 95% CI 1.65–5.05). Multivariate analysis was calculated by adjusting for initial stage, Eastern Cooperative Oncology Group Performance Status (ECOG PS) and SUV_MV_ categorized by 45 cm^3^. The results of this analysis exhibited the same tendency in HR (aHR) for PFS of EGFR TKI (Table 2). However, the aHR for SUV_average_ was insignificant after adjustment. Representative images from subjects with short PFS and long PFS of EGFR TKI are presented in Fig 4.

**Table 2:** Cox proportional regression analysis of the optimal cutoff value calculated from the exploratory dataset.

		Number of patients		PFS			
	Optimal cutoff	Above cutoff	Below cutoff	Uni-variate analysis		Multi-variate analysis^c^^)^	
		n (%)	n (%)	HR (95% CI)	*P*	aHR (95% CI)	*P*
**Co-occurence homogeneity**	6930	17 (10.6)	144 (89.4)	3.85 (2.14–6.93)	<0.01^a^^)^	3.19 (1.47–6.92)	<0.01^a^^)^
**Voxel-alignment intensity variabillity**	692	16 (9.9)	145 (90.1)	4.57 (2.39–8.72)	<0.01^a^^)^	3.66 (1.65–8.10)	<0.01^a^^)^
**Neighborhood-intensity difference busyness**	1.97	15 (9.3)	146 (90.7)	4.39 (2.30–8.38)	<0.01^a^^)^	3.33 (1.49–7.42)	<0.01^a^^)^
**Intensity size zone** **intensity variability**	98	10 (6.2)	151 (93.8)	6.20 (2.82–13.65)	<0.01^a^^)^	4.27 (1.78–10.30)	<0.01^a^^)^
**Co-occurence entropy**	-173000	153 (95.0)	8 (5.0)	6.41 (2.80–14.68)	<0.01^b^^)^	4.86 (1.97–11.98)	<0.01^b^^)^
**Voxel-alignment short zone emphasis**	0.43	140 (87.0)	21 (13.0)	4.50 (2.42–8.39)	<0.01^b^^)^	3.95 (1.77–8.81)	<0.01^b^^)^
**Neighborhood-intensity difference contrast**	0.01192	131 (81.4)	30 (18.6)	3.09 (1.24–7.71)	0.02^b^^)^	2.37 (0.92–6.13)	0.07^b^^)^
**Intensity size zone high intensity zone emphasis**	418	146 (90.7)	15 (9.3)	3.34 (1.75–6.34)	<0.01^b^^)^	3.18 (1.53–6.55)	<0.01^b^^)^
**SUV(max)**	16.8	26 (16.1)	135 (83.9)	2.77 (1.58–4.87)	<0.01^a^^)^	2.23 (1.17–4.24)	0.01^a^^)^
**SUV(average)**	6.45	112 (69.6)	49 (30.4)	1.64 (1.01–2.70)	0.05^a^^)^	1.43 (0.84–2.41)	0.19^a^^)^
**SUV(metabolic volume)**	109	24 (14.8)	137 (85.2)	2.89 (1.65–5.05)	<0.01^a^^)^	-	-
**SUV(metabolic volume) categorized by 45cm**^**3**^	45	51 (31.7)	110 (68.3)	1.88 (1.15–3.10)	0.01^a^^)^	-	-
**Initial disease status**	-	-	-	2.01 (0.86–4.65)	0.11	-	-
**ECOG PS**	-	-	-	2.28 (1.24–4.20)	0.01	-	-
**Type of TKI**	-	-	-	0.72 (0.10–5.23)	0.74	-	-
**Type of EGFR mutations**	-	-	-	0.94 (0.74–1.20)	0.62	-	-

Initial disease status was divided into two groups: recurred and metastatic.

ECOG PS was divided into two groups: subjects with ECOG PS 0 and 1 vs. subjects with ECOG PS 2, 3, and 4.

^a)^ *P* calculated by Cox proportional regression analysis compared subjects above the optimal cutoff value to those below the optimal cutoff value

^b)^ *P* calculated by Cox-proportional regression analysis compared to subjects with below optimal cutoff value to subjects with above optimal cutoff value

^c)^ Multivariate analyses were conducted for each parameter adjusted for ECOG PS, SUV metabolic volume (categorized) and initial disease status

Abbreviations: PFS = progression free survival, HR = hazard ratio, CI = confidential interval, aHR = adjusted hazard ratio, SUV = standardized uptake value

### Incremental predictive value of textural parameters for EGFR TKI failure

When textural parameters were added to the base model, Harrell’s C-index was significantly greater than the base model in Co homogeneity (0.596 vs. 0.650; *P* = 0.02), VA intensity variability (0.596 vs. 0.644; *P* = 0.03), NID difference busyness (0.596 vs. 0.631; *P* = 0.05), and VA short run emphasis (0.593 vs. 0.669; *P* = 0.01). When a Cox regression model adjusted for both MV and textural parameters was compared to the base model, a significant incremental predictive value for PFS of EGFR TKI treatment was demonstrated in all parameters (Table 3).

**Table 3:** Incremental predictive value of textural parameters for EGFR TKI failure.

	Base model	Base model adjusted with textural parameters^a^^)^		Base model adjusted with textural parameters and metabolic volume ^b^^)^	
Harrell's C-index (95%CI)			*P* (vs. Base model)		*P* (vs. Base model)
**Co-occurrence homogeneity**	-	0.650 (0.578–0.723)	0.02	0.664 (0.582–0.745)	0.03
**Voxel-alignment** **intensity variability**	-	0.644 (0.573–0.715)	0.03	0.663 (0.582–0.743)	0.03
**Neighborhood-intensity difference busyness**	-	0.631 (0.562–0.701)	0.05	0.660 (0.580–0.739)	0.03
**Intensity size zone** **intensity variability**	-	0.630 (0.558–0.702)	0.06	0.661 (0.579–0.744)	0.03
**Co-occurrence entropy**	-	0.631 (0.559–0.704)	0.05	0.662 (0.580–0.745)	0.02
**Voxel-alignment** **short run emphasis**	-	0.669 (0.597–0.741)	0.01	0.671 (0.592–0.751)	0.02
**Neighborhood-intensity difference contrast**	-	0.632 (0.560–0.704)	0.11	0.671 (0.592–0.749)	0.04
**Intensity size zone** **high intensity zone emphasis**	-	0.640 (0.565–0.715)	0.08	0.679 (0.599–0.759)	0.01
**SUV(max)**	-	0.633 (0.562–0.705)	0.14	0.672 (0.593–0.751)	0.02
**SUV(average)**	-	0.607 (0.528–0.687)	0.61	0.664 (0.579–0.749)	0.07
**Initial disease status** **and ECOG PS**	0.596 (0.527–0.665)	-	-	-	-

^a)^ Harrell’s C-index calculated by Cox-proportional regression models adjusted for initial disease status, and ECOG PS

^b)^ Harrell’s C-index calculated by Cox-proportional regression models adjusted for initial disease status, ECOG PS, and SUV metabolic volume

Abbreviations: SUV = standardized uptake value, ECOG PS = Eastern Cooperative Oncology Group Performance Status

### Association of textural parameters with early EGFR TKI failure in validation dataset

**Fig 5:**
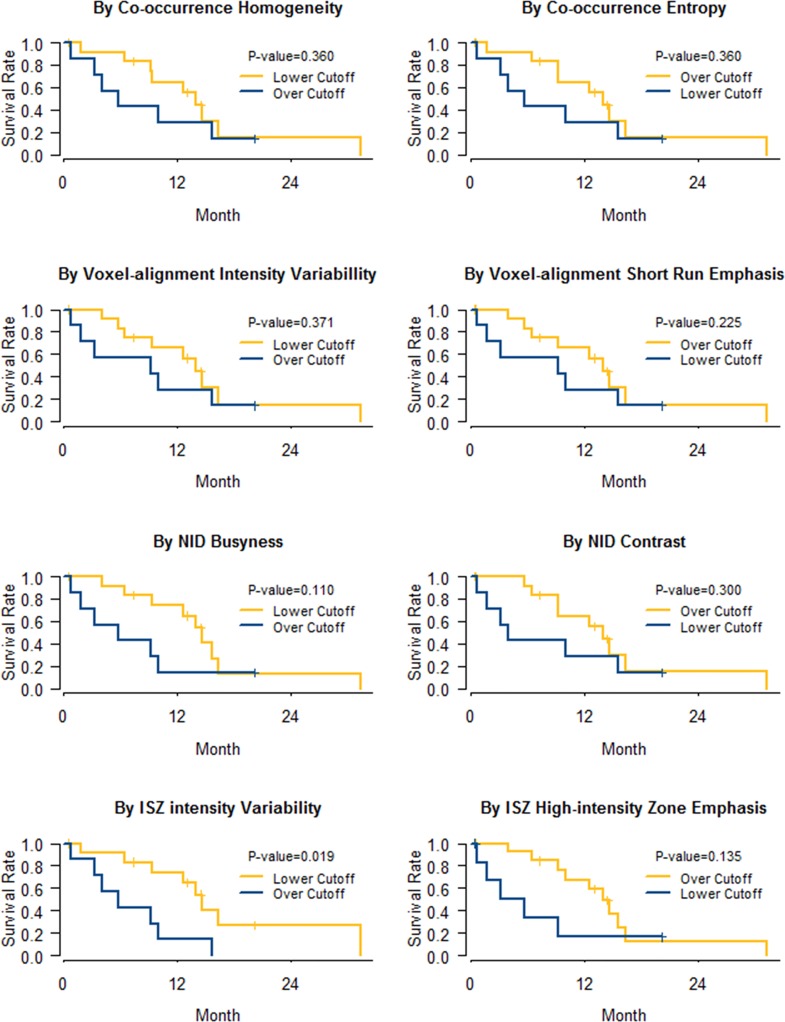
Kaplan-Meier curves of progression free survival via representative textural parameters from the validation dataset (n = 21). Binary distribution with the cutoff of the upper third value in parameter Co heterogeneity (HR: 1.65, 95% CI 0.56–4.83), VA intensity variability (HR 1.63, 95% CI 0.56–4.75), NID busyness (HR: 2.41, 95% CI 0.82–7.10), and ISZ intensity variability (HR: 3.80, 95% CI 1.24–11.6); and with the cutoff of the lower third value in parameter Co entropy (HR: 1.65, 95% CI 0.56–4.83), VA short run emphasis (HR: 1.99, 95% CI 0.66–6.02), NID contrast (HR: 1.77, 95% CI 0.60–5.18), ISZ high intensity zone emphasis (HR: 2.36, 95% CI 0.77–7.24). Abbreviations: Co = co-occurrence, VA = voxel alignment, NID = neighbor intensity-difference, ISZ = intensity size-zone.

All eight textural parameters showed increased hazard ratios with survival analysis for PFS by binary distribution at either the upper or the lower third value (Fig 5). However, only ISZ intensity variability reached statistical significance (HR: 3.80, 95% CI 1.24–11.60, *P* = 0.02).

## Discussion

In our dataset of NSCLC subjects with activating EGFR mutations, we demonstrated an independent predictive value of intratumoral heterogeneity for early EGFR TKI failure measured by textural parameters in pretreatment FDG-PET/CT. Given that a pretreatment FDG-PET/CT scan is recommended during the initial staging work-up,[30] our results have clinical implications for identifying a high-risk subpopulation for EGFR TKI treatment.

A clonal evolution model involving Darwinian natural selection has been suggested as an important cancer progression model.[31] Evidence supporting this model has been observed using next-generation sequencing (NGS) techniques, which allow the identification of genomic heterogeneity for a variety of cancers.[7, 32–34] Although a clone with an actionable mutation may be dominantly present in the trunk mutation of a tumor, a minority subpopulation with a branch mutation may contribute to treatment resistance.[35] Understanding that EGFR TKI treatment focuses on interrupting a tumor’s dependency on an *EGFR* dependent survival pathway (identified in a specific sub-clone selected by a biopsy) may result in an unidentified resistant mutant clone, such as T790M,[36] being a likely cause of treatment failure.[10] However, due to the limited representative value of a single tissue biopsy, a radiogenomic prediction model in which tumor heterogeneity is detected using metabolic activity measured by FDG-PET/CT has been suggested.[37, 38] An initial approach using standard parameters of FDG uptake was based on the hypothesis that FDG uptake shows not only factors related to metabolism, but also multiple factors related to intratumoral heterogeneity[39], especially hypoxia.^15,26^ Moreover, a genomic alteration in NSCLC was also associated with FDG uptake, and FDG uptake correlated with tumor aggressiveness and a poor prognosis of survival.[40] Therefore, approaches evolved to assess metabolic heterogeneity using textural parameters of FDG-PET/CT images, and these approaches were proved to have independent predictive value regarding treatment outcome.[15, 16, 18–20] Overall, intratumoral heterogeneity identified by metabolic texture analysis on FDG-PET/CT might be useful as a radiogenomic marker of global intratumoral genetic heterogeneity.

Conventional FDG-PET/CT parameters including SUV_max_, SUV_average_, MV, and total lesion glycolysis have been evaluated as prognostic factors for oncological treatment. [37] However, these parameters are excessively simple and are insufficient for use in combination with data from the fields of genomics, metabolomics, or proteomics. Conversely, radiomic information from FDG-PET/CT, which entails large amounts of data extracted by textural analysis, is expected to be of use in combination with genomic, metabolic, and proteomic data.[27] Nevertheless, the high correlation and dependency of each metabolic heterogeneity textural parameter and MV is an unresolved issue. This correlation and dependency are important for textural analysis as it produces complementary information for conventional parameters.[24] Controversy exists regarding the optimal MV cutoff to assure complementary information of intratumoral heterogeneity. Most of the textural parameters have a high positive correlation to MV because increased tumor size causes an increase of hypoxia and necrosis, which results in greater tissue complexity. However, this positive relationship is weakened when the tumor increases beyond a certain size. For this reason, we initially included all the data into the analysis regardless of MV. Independency and complementary characteristics of intratumoral heterogeneity features to MV were tested by multivariate analysis with categorized MV. A conservative cut off value of 45 cm^3^ was applied to MV.[41]

In this study, all subjects had documented EGFR mutations, but treatment response varied from 0.5 to 32.4 months. To validate our hypothesis, it was inevitable for authors to incorporated number of assumptions and technical approaches for the analyses. The initial approach was choosing a key representative marker. Co entropy was chosen in this study based on previous analyses which demonstrated its value as a relatively representative marker of random FDG consumption on a local scale and used as a reference parameter to identify the risk population.[15]. Next approach was conducted to minimize the potential bias due to small tumor volume. Hence, metabolic volume was adjusted to exclude the possible bias from a small tumor volume by applying categorized MV with a cutoff of 45 cm^3^ to multivariate analysis.[41] Finally, validation process was conducted using a different matrix size. Due to the limited number of validation datasets, the cutoff was arbitrarily set at the upper or lower third of the data to verify that the response tendency and the risk trend was in accordance with the exploratory dataset (Fig 5).

Throughout this observation, we have demonstrated the potential predictive value of intra tumoral heterogeneity characterized by pretreatment FDG/PET-CT parameters which could provide additional value to the real clinical practice. Hence, authors carefully recommend two parameters, Co-occurrence which has demonstrated the highest HR even adjusted with ECOS PS, metabolic SUV and initial stage, and ISZ intensity variability which was statistically significant in validation dataset, as an initial approach to predict the response of EGFR TKI through FDG/PET-CT.

This study has some limitations. It was retrospectively designed and the statistical power is insufficient due to the limited number of subjects in the validation dataset. However, we specified the criterion for the inclusion population with a comprehensive review of clinical data. A limited population limits the ability of statistical analysis to determine the optimal cutoff value and appropriate textural parameters. In addition, the validation dataset was acquired from a population with a different matrix size. Our hypothesis was based on previous studies which reported that tumor heterogeneity can be visualized with radiologic imaging.[27] In order to confirm a correlation to genomic heterogeneity, each genomic profile from multiple biopsies conducted on a single tumor mass should be directly compared to the textural parameters acquired from radiological imaging. However, considering that target therapy is performed on candidates unsuitable for surgical resection, acquiring multiple samples for validation is impracticable. Last but not least, FDG-PET/CT has a fundamental limitation as a tailored predictive modality since its image reflects various tissue reactions which could weaken a representative value of our textural parameters. [42]

## Conclusions

Our study indicates that pretreatment metabolic textural parameters can be used as predictive markers for PFS of EGFR TKI in NSCLC with an activating EGFR mutation. Pretreatment metabolic heterogeneity should be more carefully evaluated and subjects with increased metabolic heterogeneity should be considered as a high-risk subpopulation for early EGFR TKI failure. Future studies should evaluate any correlation to genomic alteration.

## Supporting information

S1 Fig **Hazard ratios of textural parameters for tyrosine kinase inhibitor progression free survival: Binary distribution at cutoff value of (A) upper 10%; (B) upper 25%; (C) lower 10%; (D) lower 25%.** Abbreviations: Listed in S1 Table.
(TIF)

S1 Table Abbreviation and definition of each texture parameter, and spearman coefficient with co-occurrence entropy. (DOCX)

S2 Table Raw data used for the analysis. (XLSX)
