# Supplementary figures and images for: Intratumoral heterogeneity characterized by pretreatment PET in non-small cell lung cancer patients predicts progression-free survival on EGFR tyrosine kinase inhibitor
Source: PLoS One. 2018 Jan 31;13(1):e0189766. doi: 10.1371/journal.pone.0189766 (PMC5791940; doi:10.1371/journal.pone.0189766)

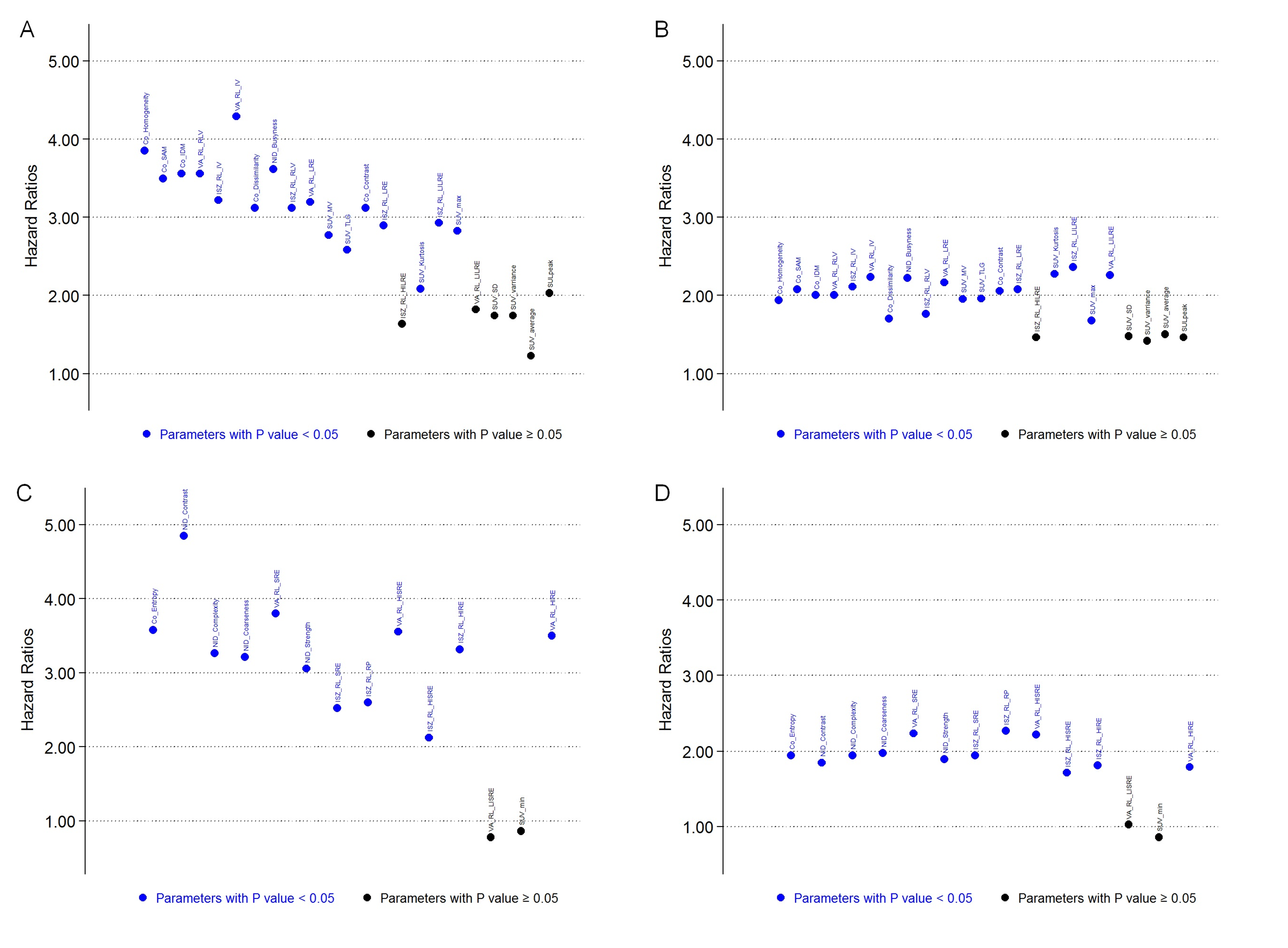

Supplement: S1 Fig — Hazard ratios of textural parameters for tyrosine kinase inhibitor progression free survival: Binary distribution at cutoff value of (A) upper 10%; (B) upper 25%; (C) lower 10%; (D) lower 25%. Abbreviations: Listed in S1 Table. (TIF) [file pone.0189766.s001.tif]
